# TuberQ: a *Mycobacterium tuberculosis* protein druggability database

**DOI:** 10.1093/database/bau035

**Published:** 2014-05-07

**Authors:** Leandro Radusky, Lucas A. Defelipe, Esteban Lanzarotti, Javier Luque, Xavier Barril, Marcelo A. Marti, Adrián G. Turjanski

**Affiliations:** ^1^Departamento de Química Biológica, Facultad de Ciencias Exactas y Naturales, Universidad de Buenos Aires, Pabellón II, Buenos Aires C1428EHA, Argentina, ^2^INQUIMAE/UBA-CONICET, Facultad de Ciencias Exactas y Naturales, Universidad de Buenos Aires, Pabellón II, Buenos Aires C1428EHA, Argentina, ^3^Department of Physical Chemistry, Faculty of Pharmacy and Institute of Biomedicine (IBUB), University of Barcelona, Campus de l'Alimentació Torribera, Avgda. Prat de la Riba 171, Santa Coloma de Gramenet 08921, Spain, ^4^Department of Physical Chemistry, Faculty of Pharmacy and Institute of Biomedicine (IBUB), University of Barcelona, Avgda. Diagonal 643, Barcelona 08028, Spain and ^5^Catalan Institution for Research and Advanced Studies (ICREA), Passeig Lluís Companys 23, Barcelona 08010, Spain

## Abstract

In 2012 an estimated 8.6 million people developed tuberculosis (TB) and 1.3 million died from the disease [including 320 000 deaths among human immunodeficiency virus (HIV)-positive people]. There is an urgent need for new anti-TB drugs owing to the following: the fact that current treatments have severe side effects, the increasing emergence of multidrug-resistant strains of *Mycobacterium tuberculosis* (*Mtb*), the negative drug–drug interactions with certain HIV (or other disease) treatments and the ineffectiveness against dormant *Mtb*. In this context we present here the TuberQ database, a novel resource for all researchers working in the field of drug development in TB. The main feature of TuberQ is to provide a druggability analysis of *Mtb* proteins in a consistent and effective manner, contributing to a better selection of potential drug targets for screening campaigns and the analysis of targets for structure-based drug design projects. The structural druggability analysis is combined with features related to the characteristics of putative inhibitor binding pockets and with functional and biological data of proteins. The structural analysis is performed on all available unique *Mtb* structures and high-quality structural homology-based models. This information is shown in an interactive manner, depicting the protein structure, the pockets and the associated characteristics for each protein. TuberQ also provides information about gene essentiality information, as determined from whole cell–based knockout experiments, and expression information obtained from microarray experiments done in different stress-related conditions. We hope that TuberQ will be a powerful tool for researchers working in TB and eventually will lead to the identification of novel putative targets and progresses in therapeutic activities.

**Database URL:** http://tuberq.proteinq.com.ar/

## Introduction

According to the last World Health Organization global tuberculosis (TB) report, in 2012 an estimated 8.6 million people developed the disease, leading to 1.3 million deaths [including 320 000 among human immunodeficiency virus (HIV)-positive people] (1). Common therapeutics for TB involves a long treatment with the front-line drugs, isoniazid, rifampicin, pyrazinamide and ethambutol (2). However, the emergence of multidrug-resistance and extensively drug-resistance (MDR and XDR) strains of *Mycobacterium tuberculosis* (*Mtb*), and the negative drug–drug interactions with certain HIV (or other disease) treatments, revealed the urgent need for new anti-TB drugs (3, 4). Knowledge of the *Mtb* genome, which comprises around 4000 genes, opened new avenues to disclose novel therapeutic approaches to TB (5–8). In particular, the analysis of the genome has the potential to extract information valuable for developing new therapies and interventions needed to treat this disease. In recent years several databases have appeared that integrate genome details, variation, protein information and transcriptome of *Mtb*, such as Tuberculist, tbvar, TBDB or TDR-Targets (5–8). In this context, the main emphasis of this work is to offer information based on recently reported structure-based predictors of protein druggability that might be valuable for target selection in drug design projects (9).

Druggability is a concept used to describe the ability of a given protein to bind a drug-like molecule, which in turn modulates its function in a desired way (10, 11). From a purely structural point of view, it can be related to the likelihood that a small molecule binds a given protein target with high affinity (<1 μM), a concept also referred as bindability, although the latter does not take into account the drug-likeness of potential ligands (12).

First attempts to determine the druggable genome of an organism, based on counting the number of targets belonging to domains known to be druggable, yielded values in the 10–14% range for the human genome (10). Similar approaches were used to identify potential drug targets in *Mtb* (13, 14), but none of them performed a whole *Mtb* proteome structural assessment. Druggable proteins should have a pocket with suitable features that enable binding of a drug-like compound (11, 15, 16). Recently, we developed a fast method for druggability prediction based on the open-source pocket detection code fpocket, which combines several physicochemical descriptors to estimate the pocket druggability and can be used on a genomic scale (9). Accordingly, fpocket was adopted as the starting point to build a whole-genome *Mtb* protein druggability database.

Antibacterial drugs exert their biological effect in a given physiological condition. To include this property we incorporated information related to the essentiality of each gene-protein, which thus when inhibited, would result in bacteriostatic or bactericidal effects (7). Essentiality of *Mtb* genes relies on experimental mutagenesis assays (17–19), *in silico* studies based on flux balance analysis of metabolic pathways (20, 21) and the determination of metabolic choke points (22). Regarding the relevance of potential targets in the pathological state, several works in the past decade have looked for *Mtb* pathogenicity-related genes using mainly genome-wide DNA microarrays in a variety of conditions, which are supposed to mimic some aspects of the environment encountered by the bacillus inside the macrophage (18, 19, 23–27). TuberQ incorporates extensive information related to the essentiality and reported expression under stress conditions using manually curated literature data. Last but not least, to perform an inhibitory effect, drugs usually target an enzyme active site, a feature that must also be considered in relation to the druggability of a given pocket.

To contribute to the quest of new antitubercular drugs from a target point of view, in the present work we generated a whole-genome *Mtb* protein database, named TuberQ, that relates structural druggability analysis of all previously solved *Mtb* proteins and new generated models with the features of putative drug binding sites, eventually compiling information derived from drug binding pockets in similar proteins, as well as information about gene essentiality, expression levels under different conditions, relevance and off-target criteria. Overall, TuberQ affords a whole-genome *Mtb* protein druggability database that incorporates structural information of previously solved *Mtb* structures and models obtained by our comparative modeling pipeline together with their structural druggability, essentiality, gene relevance and off-target criteria. The combination of structural (druggability) and physiological (essentiality) information makes TuberQ a useful tool, for example, for discarding genes that appeared to be good targets based on its biological relevance, but without relevant druggable pockets, or in discovering new druggable pockets, including allosteric sites, in already known targets. Altogether, the database allows a simple and fast inspection of protein structures and pocket druggability in the context of the available experimental information regarding the relevance of the protein for bacterial survival.

## Data set and methods

### General concept

**Figure 1.**
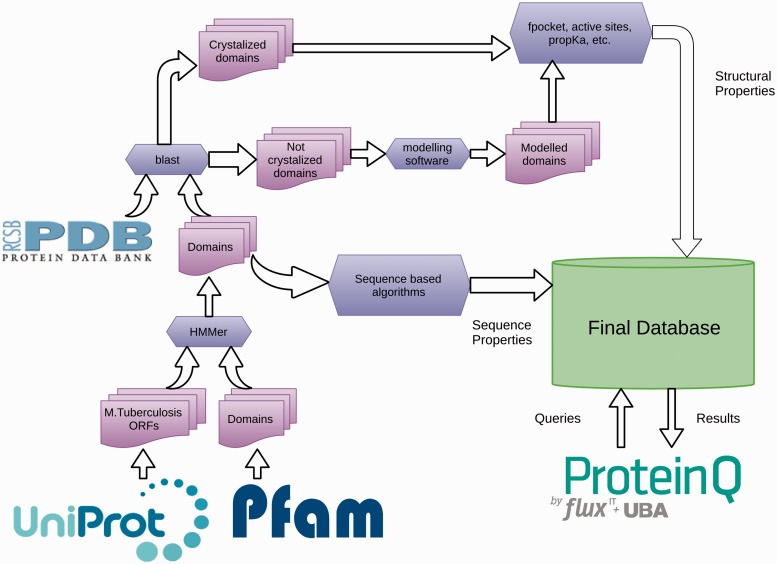
A schematic representation of the TuberQ Pipeline.

The TuberQ pipeline consists of the following steps (Figure 1). The *Mtb* Open Reading Frame (ORFs) sequences and associated metadata are downloaded from the UniProt database (28). All ORFs are then analyzed with the HMMer software (29) and the structural domains are assigned. Then, each ORF is used to perform a BLAST search (30) against the Protein Data Bank **(PDB)** (31) to determine whether the structure of the ORF (or some part of it) has been solved. Based on these results, each ORF (or domain) is classified as Solved or Unsolved. The structure of Unsolved ORFs (or domains) is modeled according to our pipeline if a suitable template is available. For all the 3D (experimental and *in silico*) structures**,** several structural properties are computed, including (i) the **druggability score** (DS) for each pocket, (ii) the similarity with human protein (to evaluate potential off-target effects), (iii) the active site residues (if available), (iv) the conserved or family relevant residues and (v) the potential sensitivity to reactive nitrogen/oxygen species (RNOS) due to the presence of specific residues/cofactors in the active site. This information is then combined with the essentiality criteria and expression**-**related information with the pipeline-engine ProteinQ.

A detailed description of the programs and databases used to perform each of the aforementioned pipeline steps is given below.

#### Downloading of Mtb protein sequences

All ORFs or possible proteins from *Mtb* H37Rv, as derived from the complete genome sequencing (32), were downloaded from the UniProt database (www.uniprot.org, organism code 3A1773) (28). This results in 3982 ORFs.

#### PFAM domain assignment

All ORFs were analyzed with the HMMer program (29) and assigned to PFAM families or domains, leading to 5822 domain assignments to PFAM-A, 1446 domains to PFAM-B and 1255 ORFs with no domain assigned. The number of ORFs with a domain assigned is 1920. However, as expected, more than one ORF can be assigned to the same domain. Thus, considering this information we could assign 1658 unique (i.e. different) domains in the whole *Mtb* genome. On average, *Mtb* genome has 2.13 domains per ORF and 1.19 unique domains per ORF.

#### Loading of microarray expression data under stress conditions

To determine which targets are relevant under stress conditions, we carried out a combined analysis of multiple published gene expression data sets derived from microarray experiments performed under a variety of conditions that model different suspected aspects of the dormant state. Given the lack of a detailed knowledge of the real physiological conditions in the dormancy phase, several studies have developed models mimicking this state, such as hypoxia, starvation and macrophage culture among others (23, 33). To the best of our knowledge, this is the most updated and complete set studied so far, and represents an update of the analysis performed by Murphy and Brown in 2007 (33).

#### Essentiality criteria

We included four available whole *Mtb* genome essentiality criteria. Rubin and coworkers performed a series of studies using a genetic technique known as Transposon Site Hybridization (TraSH), where a random insertion of this mobile genetic element is made to knockout a gene (17–19). This technique was used in an *in vitro* culture study (18), and the resulting library was subsequently used in a C57BL/6J mouse model to determine the relative abundance of the different *Mtb* genetic lines. From this work 192 genes predicted (*P* < 0.005) to be essential *in vivo* were added to our database. In the third study, a macrophage survival analysis was performed using the same TraSH library (19). Finally, in the fourth study, Sassetti and coworkers used a himar1-based transposon mutation system to determine the frequency of insertions, thus providing an update to the previous works by Rubin and coworkers (34).

#### Generation of structural homology-based models

Up to now, 441 unique X-ray structures are available for *Mtb* proteins in the PDB. For all remaining ORFs, we attempted to build homology-based models using the following structural genomic pipeline. For all *Mtb* ORFs, the first step consists in performing a psi-blast search against a template library, which includes all sequences from every individual protein chain in the PDB, grouped at 95% sequence identity threshold using CD-hit (35). Then, every target structure was built with the MODELLER software (36), using local alignment derived from the above-described psi-blast search (37). For each target sequence, 10 different models were built, and their quality measures were assigned using the GA341 (38) and QMEAN (39) methods. Only those models with GA341 score above 0.7, QMEAN between −2 and 2 and over 60% coverage were retained. This procedure yielded 903 high-quality structural homology-based models, which comprised over 34% of all *Mtb* ORFs*.*

#### Structural assessment of druggability

**Figure 2.**
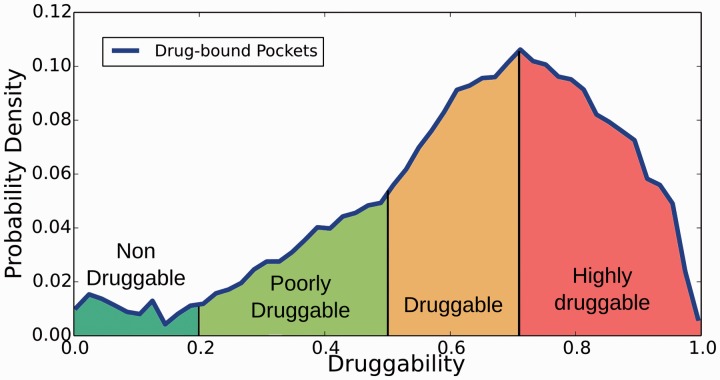
Distribution of pocket in *Mtb* proteins according to the classification derived from the DS index.

Structural druggability of each potential target was assessed by determining (and characterizing) the ability of putative pockets to bind a drug-like molecule by using the fpocket program (40) and the recently developed DrugScore (DS) index (9). Briefly, the method is based on Voronoi tessellation algorithm to identify pockets and computes suitable physicochemical descriptors (polar and apolar surface area, hydrophobic density, hydrophobic and polarity score) that are combined to yield the DS, which ranges between 0 (nondruggable) to 1 (highly druggable). Based on a preliminary analysis of DS distribution for all pockets that host a drug-like compound in the PDB (see Supplementary Figures S1–S4 for more details), in relation to other less druggable or undruggable pockets, pockets are classified in four categories (Figure 2): (i) nondruggable (ND; DS ≤ 0.2), (ii) poorly druggable (PD; 0.2 < DS ≤ 0.5), (iii) druggable (D; 0.5 < DS ≤ 0.7) and (iv) highly druggable (HD; DS > 0.7). The analysis is presented as additional information on the TuberQ Web site and briefly discussed in the present manuscript.

Taking into account oligomerization, for each protein that has been solved as a protein complex, we have added all the structural druggability information relative not only to the subunit or the monomer but also to the complex, which would allow the possibility to look for druggable pockets at the protein interface and thus enable the development of drugs targeting protein–protein interactions. Finally, to take into account possible issues related to protein flexibility, when available, we have computed the druggability of pockets for all available 3D structures of the same protein.

#### Active site identification

To identify the active site pocket and/or determine the relevance of a given pocket to protein function, TuberQ implements two different analyses that rely on (i) the information from the CSA (Catalytic Site Atlas, 41) and (ii) a PFAM position site importance criteria (42).

The data from CSA (downloaded from http://www.ebi.ac.uk/thornton-srv/databases/CSA/) consists of a list of PDB_IDs linked to a number of residues, which comprise the corresponding protein active site. To map the active sites to as many *Mtb* protein domains as possible, each PDB_ID in CSA was assigned to PFAM domains. Then, the consensus active site residues were transferred to all *Mtb* protein domains assigned to the same PFAM domains for which no CSA is available. This assignment, based on the fact that catalytic residues are expected to be conserved in a given domain, approximately doubles the amount of *Mtb* domains whose active site residues can be identified.

As an alternative approach to determine the relevance of a given pocket (or residue), we looked for residues of a given PFAM family/domain that are located in an important position and are well conserved. Important positions were defined as those positions in the corresponding HMMer model whose information content was larger than a defined importance cutoff value (*icov*). The nature of the conserved amino acids in the corresponding position was determined by comparing each residue type emission probability (*ep*) with *icov*. If the ratio between *ep* and *icov* was larger than a conserved type cutoff value (*ctcov*), the corresponding residue type was assumed to be conserved. Optimal values of *icov* and *ctcov* were 0.27 and 0.24, respectively. Further description of this methodology can be found in Supplementary Information.

By using these analyses, for each PFAM domain, TuberQ provides a list of position-residue type relevant residues, which can thus be mapped on all *Mtb* ORFs with assigned PFAM domain.

#### TuberQ updates

Updates are performed every 3 months. Updates will incorporate all new structures deposited in the PDB, as well as new models depending on the availability of the required information. We also plan to add new features in the near future, as location of MDR and XDR mutations*.*

## Results

### Description of the application

The TuberQ database can be accessed and queried using the web interface at http://tuberq.proteinq.com.ar/. The interface offers a main search menu with several options to retrieve the protein structural druggability records. The options include the use of (i) Keyword (UniProt protein name or any of the other criteria; e.g. Protein Kinase PknB), (ii) UniProt_ID (UniProtKB alphanumeric identifier; e.g. O05871 for Protein Kinase PknB), (iii) PFAM_ID (PFam family identifier; e.g. PF01436.16, NHL repeats) and (iv) PDB_ID (the PDB four alphanumerical character id; e.g. 1IDR for *Mtb* Truncated Hemoglobin N). As an example, let us assume that we already know our target protein ID. In this case, we simply type ‘P0A5Y6’ and select UniProt_ID in the scroll down menu to retrieve all associated records.

**Figure 3.**
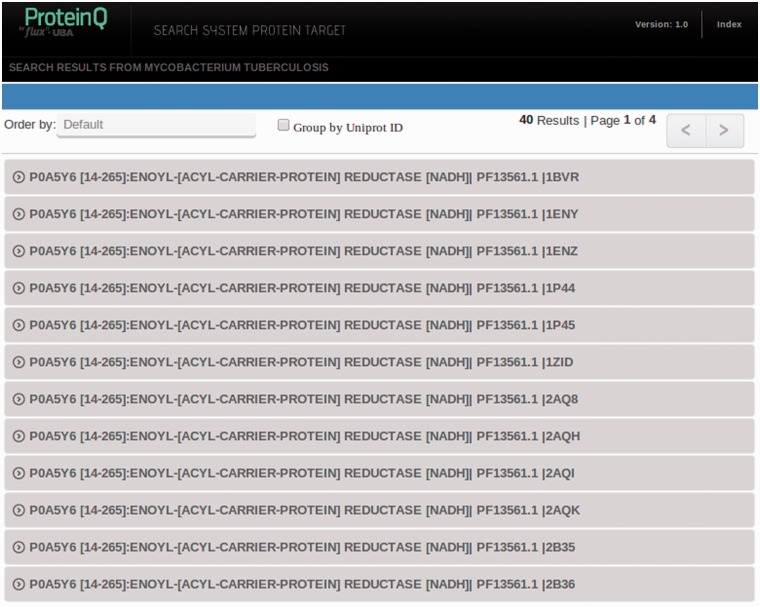
Representation of search results. Every UniProt-PFAM-structure triad represents a different entry in the database. One can choose to group entries by UniProt ID by ticking in the Group by UniProt ID box.

Searches may return a single database entry (e.g. when searching by PDB_ID or UniProt_ID) or multiple entries (e.g. Keyword and PFAM_ID searches). The resulting records are listed in the search results page (shown in Figure 3) and can be ordered by ascending or descending DS. For each record, the UniProt_ID, protein ‘common’ name, PFAM domain and the PDB_ID or homology-based model ID are presented. In the example, our protein of interest has been crystallized several times, and for each X-ray structure, one can find the corresponding structural druggability record in the database. By right clicking on the desired row, the information of the corresponding record will be expanded.

For each record, three main tabs (always accessible on the left side of the screen) can be displayed. In the Initials-Tab, shown in Supplementary Figure S5, protein general information and data are presented, together with the assignment (and corresponding links) to a given PFAM protein family and structure. In the current example, UniProt_ID P0A5Y6 is assigned in almost its whole length to PF1356, which corresponds to ‘Enoyl (Acyl Carrier Protein) Reductase’ domain. The proteins also matched several structures deposited in the PDB. For our example, we will further analyze the structure corresponding to PDB_ID 2NV6 (see below). Also, in the Initials-Tab, the best hit against the human genome obtained using the blast program is also shown.

Selecting any of the **Structure-Tabs**, by clicking on the PDB_IDs shown at the left side of the screen, presents the structure-related data, including the interactive pocket visualization module (see Supplementary Figure S6). The visualization module allows the user (i) to select a given pocket for graphical display (by ticking the corresponding pocket Select field), (ii) to display present HETATOMS, assigned CSA or PFAM-relevant residues, (iii) to display the protein as chain, bonds or sticks and (iv) to display the pocket residues or the alpha spheres defining the pocket. In the example shown below, we depict the alpha spheres of pocket ‘0’ in green, which is a HD pocket, the HETATOMS found in the crystal structure as spheres and the protein as ribbons. Another visualization of the same pocket could be to show the residues lining the pocket (instead of the alpha spheres), and the residues reported to be part of the active site to see if some of them match relevant residues (see Supplementary Figure S7). The displayed protein can be downloaded as a compressed file for both VMD and PyMol (43, 44) programs. Inside this file, two scripts (xxxx_VMD.sh and xxxx_PyMol.sh, where xxxx corresponds to the structure identifier) allow the user to display graphically the protein with the desired software.

Additional information is provided at the bottom of the Structure-Tab. For example, details of the crystallized ligand (in the example, the ZID ligand) can be obtained by right clicking on the ligand. Complete information on all pockets identified in the protein by the fpocket software is also accessible by right clicking on ‘Pockets’ at the bottom of the page. The corresponding page shows all the pockets ordered by their DS, together with other pocket parameters, whereas only those pockets that have been classified as D or HD (see above) are shown in the Structure-Tab. Finally, in the third tab, the Metadata tab, information related to other databases (such as UniProtKB) and literature is displayed. In our example, sites on the protein sequence depicted in UniProtKB as nucleotide binding sites are shown along with a report in which the protein is described as not essential. Moreover, in this tab, expression profile of the chosen protein in various experimental settings, including exposure to NO and H_2_O_2_, starvation, hypoxia and expression during mice infection, is available (Supplementary Figure S8).

### Database statistics

TuberQ allowed us to analyze some interesting statistical data concerning the druggability of the *Mtb* H37Rv genome. From a pure structural viewpoint, of 1344 available structures (including X-ray structures and models, representing 34% of all *Mtb* ORFs), 82% correspond to **HD** pockets (DS > 0.7). This finding is encouraging for drug design projects, but it also may reflect the inherent bias toward the determination of ligand bound (i.e. structurally druggable) proteins in the PDB. It is important to remark that displaying a druggable pocket is a necessary, but not sufficient condition, as binding to the pocket must also modify the biological activity of the protein in the desired sense. Furthermore, evaluating the relevance of a given pocket generally demands manual inspection, as its biological effect might involve pockets other than the active site (i.e. allosteric site and protein**–**protein interaction). In this context, TuberQ offers easy inspection of pockets together with information about active site residues, PFAM**-**relevant residues or in the context of protein**–**protein complexes, besides the essentiality of the protein for bacterial survival. By combining druggability and essentiality data, among the 379 genes (9.5% of all ORFs) reported to be essential for *Mtb* growth, 352 ORFs can be identified as druggable, accounting for 8.8% of the whole genome and 26% of the structurome, and 184 as **HD** (4.6% of the whole genome and 13% of the structurome). Finally, if one also considers information about overexpression under stress conditions, which involves 713 ORFs, 145 are essential, 475 are **HD** and 111 satisfy all the criteria (the list of best candidates is presented in Supplementary Table S1).

### Highlighted examples

Researchers approaching TuberQ may be interested in different aspects of the database. For example, if one looks for essential proteins or overexpressed proteins in stress conditions, it can be found that there are 11 proteins that are described as essential for growth and/or infection in *Mtb* that have been classified as ND or PD (DS < 0.5) (Supplementary Table S2) and 29 proteins that are overexpressed and are ND or PD. As an example, phosphoribosyl-ATP pyrophosphatase is an essential protein (17) and is highly overexpressed during RNOS stress (23), which make it a very attractive target for drug design. Nevertheless, from a structural point of view the protein is PD, as the pockets are found to be superficial and small, thus rendering them not suitable for drug design.

Another interesting feature is the structural mapping of important PFAM residues together with druggable pockets. This can be useful to highlight important residues for protein function when active site or binding site data are not available. As an example, we quote the case of Universal stress protein Rv1636/MT1672 (O06153), which has been crystallized in its apo form. This protein has a small but HD pocket, which contains important PFAM family residues, which may be attractive for mutational studies aimed at molecular and functional characterization of the protein. Moreover, Rv1636 has been shown to be upregulated in NO/H_2_O_2_ stress conditions (23), making it an attractive candidate for further exploration as a drug target.

### Comparison with other available resources focusing on druggability

In the **p**ast decade**,** several computational methods have been developed for determining the druggability of a protein (45). Most of them rely on cavity detection algorithms to identify pockets, and use several physical**-** and/or chemical**-**based descriptors to make their prediction. The fpocket program used in TuberQ belongs to this group. The main differences between the predictors usually rely in the set of **D** and **ND** structures adopted to train the method (for example, only those structures with ligands that are drugs known to be orally available) and the specific subset of all possible pocket descriptors that were considered. The general trend shows that most of them have reached a fair level of predictive power, with success rates for positive site detection in the 70–90% range (9, 12, 46–52). It is important to note, however, that **because** most of these methods rely solely on structure and the identified pocket properties, hits are usually more indicative of bindability rather than its druggability (see above). Also, most of them are programs that need to be downloaded, installed and run locally by the researcher for a given target, or group of targets, thus requiring some expertise to obtain the prediction. To the best of our knowledge, so far only the DoGSiteSCorer method has been made available through a **W**eb server (53).

In this context, TuberQ takes advantage of the structure-based druggability prediction methods (fpocket) and provides information about druggability by classifying the pocket in one of four simple categories, which would facilitate the user to evaluate the DS results (Figure 2). Furthermore, TuberQ combines the results with biological metadata that allows direct evaluation of the potential therapeutic impact of the target. Moreover, data are already computed and directly available for the researcher (even for downloading), making the present resource, to the best of our knowledge, unique in the mentioned issues. It is worth noting that our whole-genome comparative modeling pipeline allowed the inclusion of more than 900 new structures, which can be visualized and compared with available X-ray structures and will allow users to evaluate proteins for which structural information was not available.

On the other hand, due to the relevance of *Mtb* and the potential of whole-genome target identification approaches after deciphering of its genome (32), several *in silico* based works have appeared on the subject (7, 21, 22, 54–56). In few instances, they considered some druggability prediction (including in some cases structural aspects), a role in dormancy based on gene expression data, essentiality and off-target criteria to avoid potential unwanted side effects. However, they tend to end with a list of potential ‘best’ targets, which are presented as a closed case. None of them is interactive or allows the user to analyze and weigh the data based on her/his own criteria. Our database has been designed to offer these possibilities, as it was conceived as a tool to assist the decision-making process in *Mtb* drug development through an interactive and regularly updated framework. TuberQ offers a wide range of diverse applications. For example, searching for bindability in our database could help deciding the suitability of a protein target, or alternatively a researcher may be interested in looking for specific protein functions and find all the metadata combined with bindability and location of pockets for selection of the most promising targets.

## Conclusions and perspectives

In this work we have combined most of the information related to *Mtb* protein relevance and sensitivity, essentiality and off-target criteria with structural druggability prediction and analysis in a user-friendly database, with graphical facilities for structural visualization and manipulation. We believe that this database is highly useful for people working in the field of drug discovery, target selection and structural biology of TB. TuberQ is the first database to provide a comprehensive analysis of *Mtb* genes structure and pocket identification with a DS. In our database, users can easily find if a desired target, selected perhaps by relevance, has a druggable pocket and is therefore worth continuing the development of new drugs. We plan to extend the present analysis to include information concerning the molecular basis of MDR and XDR, and their potential relation to druggability issues, links and scores related to other Drugs-for-TB–related databases, such as the TB drugome database (14) and links and information related to TB genome variation like Tbvar (57) Finally, we believe our database shows interesting features from a bioinformatics perspective, as there are few databases that combine structure-based druggability with functional and physiological data at a whole-genome level. Finally, the druggability pipeline strategy outlined here will in the near future be extended to other pathogens, especially those causing the so-called ‘neglected diseases’.

## Supplementary Data

Supplementary data are available at *Database* Online.
